# **R**enal **A**thersosclerotic re**V**ascularization **E**valuation (RAVE Study): Study protocol of a randomized trial [NCT00127738]

**DOI:** 10.1186/1471-2369-8-4

**Published:** 2007-01-26

**Authors:** Sheldon W Tobe, M Atri, N Perkins, R Pugash, Chaim M Bell

**Affiliations:** 1 Departments of Medicine and Radiology, Sunnybrook Health Sciences Centre, University of Toronto, Toronto, Canada; 2 Department of Medicine, St. Michael's Hospital, Departments of Medicine and Health Policy Management and Evaluation, University of Toronto, Toronto, Canada

## Abstract

**Background:**

It is uncertain whether patients with renal vascular disease will have renal or mortality benefit from re-establishing renal blood flow with renal revascularization procedures. The RAVE study will compare renal revascularization to medical management for people with atherosclerotic renal vascular disease (ARVD) and the indication for revascularization. Patients will be assessed for the standard nephrology research outcomes of progression to doubling of creatinine, need for dialysis, and death, as well as other cardiovascular outcomes. We will also establish whether the use of a new inexpensive, simple and available ultrasound test, the renal resistance index (RRI), can identify patients with renal vascular disease who will not benefit from renal revascularization procedures[1].

**Methods/design:**

This single center randomized, parallel group, pilot study comparing renal revascularization with medical therapy alone will help establish an infrastructure and test the feasibility of answering this important question in clinical nephrology. The main outcome will be a composite of death, dialysis and doubling of creatinine. Knowledge from this study will be used to better understand the natural history of patients diagnosed with renal vascular disease in anticipation of a Canadian multicenter trial. Data collected from this study will also inform the Canadian Hypertension Education Program (CHEP) Clinical Practice Guidelines for the management of Renal and Renal Vascular Disease. The expectation is that this program for ARVD, will enable community based programs to implement a comprehensive guidelines based diagnostic and treatment program, help create an evidence based approach for the management of patients with this condition, and possibly reduce or halt the progression of kidney disease in these patients.

**Discussion:**

Results from this study will determine the feasibility of a multicentered study for the management of renovascular disease.

## Background

End-stage renal disease (ESRD) comprises an enormous public health burden, with an incidence and prevalence that are increasing alarmingly[2]. The two most common causes of ESRD are diabetic nephropathy and hypertension while ARVD is the most rapidly increasing cause. The prevalence of ARVD is 2% of those with essential hypertension rising in those with severe hypertension or resistant hypertension to 20–40%[3,4]. There is no preferred, evidence based pathway for the diagnosis and management of ARVD, and the clinical practice guidelines describing the investigation and treatment of this condition are complex and based to a great extent on opinion[5,6]. Some of the diagnostic tests are very expensive. The therapeutic procedure for treating the condition, revascularization, is invasive, expensive, particularly if a stent is used, and it is not yet clear whether revascularization helps to preserve or restore kidney function.

### Atherosclerotic renovascular disease

ARVD occurs from progressive atherosclerosis of the arteries of the kidneys as a result of the effects of factors such as high blood pressure, diabetes, elevated cholesterol and smoking on the blood vessel walls. Narrowing of the large and/or small arteries of the kidneys leads to renal ischemia. Affected areas of the kidney, responding to progressively lower blood flow rates, try to restore their blood flow by producing renin leading to elevated levels of angiotensin II and aldosterone. These hormones cause systemic hypertension, aggravating the atherosclerotic process and completing a positive feedback loop[7]. Higher blood pressure further damages the arteries of the kidneys causing renal ischemia, fibrosis and progressive loss of renal mass and renal function, which is also a stimulus for higher blood pressure. ARVD typically occurs in an older population group, with risk factors for and evidence of, generalized atherosclerosis. Fibromuscular dysplasia, a cause of renal artery stenosis that presents at a much younger age, is not the subject of this study and patients with this condition will be excluded through the inclusion and exclusion criteria.

A recent meta analysis demonstrated that revascularization of the renal artery in patients with > 50% narrowing, resulted in only a small improvement in blood pressure control when compared to medical management[8]. In addition, no benefit in renal outcomes were found. The studies in the meta-analysis focused on treating anatomical lesions rather than focusing on the recognition and prevention of intrarenal parenchyma damage. This damage to the nephrons, the filtering units of the kidney, has been found to be more important in the pathogenesis of renal dysfunction than the presence of renal artery stenoses > 50% of the vessel lumens[9]. Renal protection strategies which include blood pressure control, the use of aspirin and cholesterol reduction with statins are part of the medical management to prevent renal parenchyma disease[10]. Revascularizing lesions of the renal artery greater than 50%, may help to improve blood flow to affected areas of the kidney. However, if the affected areas have already suffered renal parenchyma damage, then restoring blood flow will not result in an improvement in renal function. Thus a diagnostic strategy that could effectively identify patients who will not benefit from revascularization could save patients from invasive and costly tests which will not benefit them.

### Diagnosis of ARVD

Early diagnosis and treatment of ARVD may help to improve hypertension and prevent renal impairment. The current diagnostic recommendations from the Canadian Hypertension Education Program clinical practice guidelines can be found in Table 1. Clinically, signs of renal artery stenosis include; abdominal bruit, resistant hypertension requiring three or more anti-hypertensive medications, accelerated or suddenly uncontrolled hypertension and atherosclerosis in other vascular beds. Stenoses less than 50% are not associated with renal impairment or hypertension[11], but those greater than 50% can cause both[7]. Early onset of hypertension before the age of 30 suggests fibromuscular dysplasia, an as yet idiopathic form of renal artery disease and not the subject of this study. Sudden onset of resistant hypertension after the age of 55 years suggests ARVD as does differences in the size of both kidneys greater than 1 cm, on ultrasound. These clinical signs can help identify a patient with increased likelihood of disease. Once ARVD is suspected, a screening test should be performed to confirm its presence. One of the greatest challenges in managing ARVD is that there is no single screening test with sufficient sensitivity and specificity to confidently identify patients who will benefit from revascularization. For example while renal angiography can identify whether there is macrovascular renal artery disease which is amenable to percutaneous transluminal renal angioplasty (PTRA) with or without a stent, it can not determine if the lesion is responsible for the clinical picture or predict whether revascularization will improve blood pressure control or preserve renal function. Angiographic procedures may cause renal harm by introducing contrast dye directly into the kidney as well as introducing athero-emboli by disrupting atheroma along the vessel wall[12]. While revascularization studies have demonstrated good anatomical correction of renal vascular lesions, particularly with renal stenting (which costs many thousands of dollars)[13], even meta analyses of studies of clinical revascularization have shown no improvement in renal function compared to conservative strategies and modest if any improvement in blood pressure control[8]. In general, studies of PTRA without or with stenting (PTRAS) for ARVD have shown that renal function is improved in about 25%, stabilizes in about 40%, but worsens in about 25% of patients[14].

**Table 1:** Clinical clues to suggest the presence of atherosclerotic renovascular disease

Clinical clues
• DBP >95 or ISH with uncontrolled BP despite 2 drugs.
• Declining GFR – Creatinine rises >20% with ACEI/ARB or >20% rise over 1 year without glomerulonephritis or diabetes.
• Hypokalemia with hypertension, high renin.
• Abdominal bruit.
• Flash pulmonary edema.
• Peripheral vascular disease and other atherosclerotic manifestations -coronary artery disease, cerebrovascular disease.
• Atherosclerotic risk factors – older age (> 55), smoking, hyperlipidemia, diabetes, hypertension, male gender.

### Diagnostic screening tests

The ideal single screening test for ARVD would be accurate, have low technical failure, high sensitivity and specificity and it should be non-invasive and inexpensive[1,15]. Magnetic resonance imaging (MRI) and spiral CT are non-invasive and have high sensitivity and specificity but have high costs and for spiral CT include the risk for contrast dye. MRI is also contraindicated for patients with claustrophobia, metallic implants or for the seriously ill. Simple measurements of serum renin have been investigated to predict the potential success of surgical revascularization but the frequency of false-negative and false-positive results make this screening test unhelpful[16]. Even when the accuracy of the serum renin test is enhanced by the addition of an ACEi (angiotensin converting enzyme inhibitor) the test still has insufficient specificity and sensitivity to be recommended[17]. Also, renin based diagnostic tests are limited by antihypertensive drugs that interfere with plasma renin activity.

The captopril renal scan can detect ARVD with high sensitivity and reasonable specificity and also gives information on the potential for blood pressure lowering following revascularization[11]. This test is limited in that it cannot locate the stenosis or determine its severity and it has not been shown to predict improvements in renal function[18]. Also the sensitivity of this test is reduced in patients with renal insufficiency or bilateral stenoses or a single kidney with vascular stenosis, clinical scenarios common enough to exclude this test alone as a screening tool[19].

Doppler ultrasonography has been demonstrated to have high sensitivity and specificity in experienced hands for the diagnosis of anatomical lesions[20]. It can also detect unilateral and bilateral lesions, accessory renal artery stenosis, is non-invasive and is not expensive. Obesity, excessive bowel gas or poor blood flow in the main renal artery can interfere with direct visualization. The RRI performed as part of a Doppler ultrasound exam is a powerful tool for identifying patients that may not benefit from revascularization. It is easy to learn and can effectively be applied to all patients referred for a simple abdominal ultrasound as part of the initial investigation for ARVD.

Preservation of renal function and blood pressure control are the benefits that make the risks of renal revascularization acceptable. Presently none of the diagnostic tests for ARVD alone are sufficient to a) determine the presence of an anatomical lesion suitable for revascularization and b) determine whether revascularization will lead to blood pressure control and renal preservation. This has led to great heterogeneity in the diagnostic investigation of ARVD and a focus on the more easily recognized and treatable anatomical disease. With no single screening test, standard of care for diagnosis of ARVD involves multiple screening tests including a renal ultrasound for renal size with Doppler analysis of the renal arteries and a captopril renal scan to determine the presence of renal artery disease and its functional significance. To confirm the diagnosis in those with intermediate or high probability of renal vascular disease, an MR angiogram rather than renal angiography is used to avoid nephrotoxic contrast and the need for the insertion of a wire into the renal artery with risk of atheroemboli. This test has high sensitivity and specificity is non-invasive and uses gadolinium for the contrast agent making it safe for patients with chronic kidney disease. In a meta-analysis, Vasbinder found that gadolinium-enhanced MRA and CT angiography had the best diagnostic accuracy even in patients with ARVD with renal insufficiency[21]. Because contrast dye is contra-indicated in many patients with ARVD due to chronic kidney disease this test is preferred over renal angiography or spiral CT.

### Renal resistance index

The RRI is a recently described test which may be able to select patients with pre-existing renal parenchyma disease that may not benefit from revascularization. This diagnostic test has not yet been tested in a randomized prospective study but has tremendous potential to help patients avoid unneeded tests. It is therefore a key component of the RAVE study.

The RRI was described by Radermacher in 2001[1]. It describes the amount of renal arterial impedance and is calculated as ((peak systolic velocity - end diastolic velocity)/peak systolic velocity) × 100 averaged over 4–6 measures in the upper, middle and lower kidney[1]. In univariate analysis it was found to have an odds ratio for detecting those who would have worsening of renal function after renal revascularization of over 100, orders of magnitude greater than the other known predictive factors[1]. Presently it is not in wide use but has great potential to exclude patients who will not benefit from revascularization. Radermacher has shown that in patients with renal artery stenosis an increase in the RRI ≥ 80% is associated with poor renal outcomes and modest if any effects on blood pressure control following revascularization consistent with micro vascular disease leading to renal parenchymal disease[1]. For example, in one study following revascularization, in subjects with RRI ≥ 80% none had blood pressure lowering, 46% required dialysis and 29% died while in those with RRI < 80%, following revascularization there was significantly lower blood pressure, 3% went on to dialysis and 3% died[1]. In both univariate and multivariate analyses, the RRI was shown to be the only reliable predictor of progression of renal disease in patients with renal artery stenosis and identified those whose blood pressure and renal function was unlikely to improve with intervention[1,22,23].

### Renal revascularization

Indications for renal revascularization include blood pressure which remains above target despite medical therapy and/or deterioration in renal function despite medical therapy. Surgery was considered the standard revascularization procedure in the past but is now a backup for PTRA because of the lower morbidity and mortality with the less invasive procedure[16]. Not every patient with ARVD will benefit from intervention and improvement may be seen in blood pressure in only 60% of unselected patients[16,24]. This falls to 25–30% in those with renal insufficiency [25]. Studies comparing revascularization to conservative therapy have never used the RRI and have reported only slight to no benefit for blood pressure control and no preservation of renal function [8, 26]. Hypertensive nephrosclerosis and diabetes as well as other clinical factors (those found in Table 2) are not sensitive or specific enough to predict those patients who are most likely to benefit from renal revascularization, or those who should not have revascularization[16].

**Table 2:** Factors associated with lower likelihood of response to renal revascularization[1]

Factors
• Urinary protein excretion ≥1 g/d,
• GFR < 40 ml/min
• pulse pressure of at least 70 mmHg
• male gender
• duration of hypertension > 10 years
• no history of smoking
• cerebrovascular disease
• hyperuricemia
• age > 65
• DBP < 80 mmHg, SBP < 160 mmHg
• no abrupt onset in blood pressure
• Diabetes mellitus
• coronary artery disease
• peripheral arterial disease

In summary, the evidence to guide selection of the patient who is most likely to benefit from renal revascularization is limited. Identifying patients most likely to benefit from revascularization is of prime importance to limit the risk of diagnostic angiography and revascularization in those who are unlikely to derive a benefit. The RRI potentially could identify patients in whom revascularization should not be considered and those in whom revascularization is more likely to be successful.

## Methods/Design

### Study aims

The primary objective of the RAVE study is to determine the frequency of progression to the composite endpoint, death, dialysis and doubling of creatinine, in patients with ARVD and an indication for revascularization, randomized to medical therapy or renal revascularization over a minimum of 6 months. The secondary objectives are to compare blood pressure and medications used in patients randomized to revascularization or medical therapy, determine the sensitivity and specificity of the RRI in identifying the response to renal revascularization, to determine baseline factors in people with ARVD that are associated with the indication for revascularization and to follow patients over time for both an intent-to-treat and per protocol analysis of outcomes stratified by their renal resistance index finding.

### Study design and setting

RAVE is a randomized controlled trial with blinding of patients, health care providers and all study staff and outcome assessors. In the study, patients with newly diagnosed renal artery stenosis from MR or CT angiography that have an indication for renal revascularization are randomized to receive either renal revascularization by PTRA with stenting or medical therapy alone.

### Ethical considerations

Ethical approval has been obtained at Sunnybrook Health Sciences Centre.

### Study interventions

The intervention is assignment to a group receiving medical therapy alone. The standard of care currently for patients with renal artery stenosis and an indication for revascularization is to have revascularization. Medical therapy includes aggressive blood pressure lowering to target (< 140/90 mm Hg and < 130/80 mm Hg for those with diabetes), LDL reduction < 2.0 with statin therapy, use of ASA or other antiplatelet therapy, diabetes control, diet, exercise and smoking cessation. Referral for diabetes education and management if appropriate.

Percuateneous transluminal renal angioplasty, involves percutaneous access to the arterial circulation using the Seldinger technique at the level of the femoral artery and the passing of an angiographic wire into the renal artery and proximal aorta for contrast dye injection. Patients are admitted to the nephrology ward the day of the procedure and are discharged the following day after lab work has been completed and reviewed. Antihypertensives are given at half dose on the day of the procedure to prevent symptomatic hypotension. ASA is held the day of the procedure. For those on coumadin, it is held 5 days before and vitamin K 1 mg administered and a normal INR is required prior to the procedure. Stenting will be performed at the discretion of the angiographer. Angioplasty of the renal artery with stenting leads to lower restenosis rates and longer term patency [27]. While it seems logical that this greater patency would lead to improved blood pressure control and preservation of renal function, no study has yet been conducted to prove this.

The choice of balloon size, angiographic method and need for stent will be determined on a case by case basis by the angiographer. In general stents are used for those patients with ostial lesions. Renal revascularization to date has not demonstrated in most patients in blood pressure control and has shown no benefit in renal preservation. The DRASTIC study, the largest study to investigate the effectiveness of a revascularization strategy for ARVD had to screen over 1200 patients referred with resistant hypertension to find 543 who received angiography for diastolic blood pressure ≥ 95 mm Hg. Of these, 169 (30%) were found to have renal artery lesions ≥ 50% [28]. The DRASTIC study did not use renal stenting and did not preselect patients using the RRI. Patients that require surgical revascularization will be withdrawn from the study.

### Identification of eligible patients

Patients referred to a renal vascular clinic in an academic health sciences center will be eligible to enter the study. This clinic is devoted to the diagnosis and management of renal vascular disease. After referral, patients have a minimum data set of screening laboratory and diagnostic tests and screening diagnostic tests including a renal ultrasound, Doppler of the renal arteries and RRI. At the baseline visit after collecting appropriate history physical examination and lab data to document the risk factors signs and symptoms of ARVD all the tests are reviewed. If the patient has an intermediate or high probability for revascularization based on a clinical assessment of the history, physical, lab and screening tests, an MR angiogram or CT angiogram is arranged. All patients receive aggressive medical management aimed at cardiovascular and renal risk reduction with a focus on blood pressure and lipid control and use of (Acetyl-salicylic acid) ASA. In this protocol, patients assessed at the baseline visit who are diagnosed with ARVD will be asked to consent to this study. Randomization to revascularization or medical management will take place later for those patients that develop indications for revascularization. All patients with renal vascular disease are followed at three month intervals. Following revascularization, patients are seen monthly for six months. Patients enrolled in the study will be receiving appropriate medical care according to clinical practice guidelines and will be considered part of regular clinical practice. No additional tests or procedures other than the consent to participate in a study, will be performed on those participating in this study.

### Randomization and study blinding

Following informed consent patients found to have renal artery stenosis will be randomized in a permuted block design with blocks of 2 and 4. The randomization schedule has been developed and will be administered by the Biomedical Design and Statistics unit. The randomization code will be kept in a sealed opaque envelope and will not be opened until the patient has an indication for revascularization or the study end is reached. The study investigators, coordinators and patients and all members of the research team will be aware of the randomization when opened but will be blinded to the results of the renal resistance index. The allocation will be administered by study nurse in sealed sequentially numbered envelopes. The study nurse will open the next number in sequence when a randomization has occurred. The date and time of randomization will be recorded and the opened randomization envelope will be filed in the trial centre binder.

### Primary outcome

The primary objective of the RAVE study is to determine the frequency of progression to the composite endpoint, death or dialysis or doubling of creatinine, in patients with ARVD stratified by the renal resistance index. Patients with an indication for revascularization will be openly randomized to medical therapy alone or to renal revascularization as well as medical therapy.

Following revascularization patients will be assessed monthly for 6 months and then every three months. All patients will have at least 6 months of follow-up. The blood pressure and creatinine level at the final visit will be used for the analysis. If an outcome is reached during the study, patients will continue to be followed, but data for the main outcome measure will be based on only the first endpoint reached. Outcomes will be analyzed on an intent-to-treat basis.

### Secondary outcome

The secondary objectives are to; 1. Compare blood pressure and medications used in patients randomized to revascularization or medical therapy, 2. determine the sensitivity and specificity of the RRI in identifying the response to renal revascularization, 3. determine baseline factors in people with ARVD that are associated with the indication for revascularization and 4. to follow patients over time for both an intent-to-treat and per protocol analysis of outcomes stratified by their renal resistance index finding.

### Data collection

Data will be collected into an Microsoft Access data base designed to capture initial visit information and generate a consultation note. Baseline data and ongoing data collection as outlined in Table 4 will be obtained.

**Table 4:** Baseline and ongoing data collection

Baseline data:
• Blood pressure will be measured at each visit using the BpTru (VSM Technologies, Vancouver, Canada)
• serum creatinine.
• U/S abdomen for renal size and Doppler presence of renal artery stenosis
• Percent function of both kidneys and change with captopril on renal scan
• MR/CT angiography to determine presence or absence of renal artery stenosis
• Med review
• allergies
• Past surgery, hospitalizations
• Initial History and physical
• Labs, urine/blood
• ECG
Follow-up lab data: every 3 months
• Urea and creatinine
• Lipids q6 months
• U/S annually for renal size

### Patient follow-up procedures

Any patient randomized to medical therapy who has an indication for revascularization will have attempted further medical management for another 3 months. Patients randomized to revascularization who have an indication will be offered revascularization. All patients will be followed prospectively for both an intent to treat and per protocol analysis. The analysis will also be carried out stratified by the result of the renal resistance index (normal vs abnormal) to determine it's prognostic ability.

The decision to proceed toward revascularization will be based on a rise in creatinine by > 20% per year from baseline for year one and in year 2, by > 20% over the average creatinine from year one. To reduce the possibility that the rise in creatinine is temporary, due for example to a hypovolemic event, a repeat creatinine will be required from 3 days to 3 months later to confirm that the creatinine has risen by 20%. If an ACEi or ARB has been started after enrollment into the study, a rise in creatinine of 20% in the first 3 months after the initiation will be permitted. The creatinine trigger for revascularization after ACEi or ARB is started will be a creatinine rise of 40% during that year. The blood pressure trigger for revascularization will be systolic blood pressure ≥ 150 mmHg and/or diastolic blood pressure ≥ 95 mmHg on any visit.

Patients randomized to the medical management group will not be excluded from revascularization. If blood pressure is ≥ 170 mm Hg and/or diastolic blood pressure ≥ 100 mm Hg on any visit despite treatment with at least three antihypertensives at maximal dosage patients can be referred for revascularization. In this circumstance, patients will continue to be assessed in an intent-to-treat analysis. If the creatinine doubles from baseline patients can be referred for revascularization.

### Study withdrawal

Patients will be censored and withdrawn from follow-up at their request. Patients will be censored at the time they reach an endpoint including the need for renal replacement therapy, death or doubling of the creatinine from baseline.

### Statistical analysis

This is a pilot study. The expected number of patients enrolled and time-frame of the study will allow an appropriate sample size calculation for a large multicentered study. The goal is to recruit 20 patients with renal vascular disease. Over the study period, 20% of these are expected to have indications for revascularization. Radermacher found that 20% of patients with ARVD had RRI ≥ 80 but it is likely that a greater fraction of those who require revascularization will have a high RRI[16]. It is estimated that this will be 1/3 in this study. This should lead to enough patients in each group to gather meaningful information to plan a larger study.

### Sample size considerations

At the time the study was planned, it was expected that the renal vascular clinic would take in an expected 240 patients over 1.5 years. During this time at least 20% of the patients are expected to have an indication for revascularization. The minimum follow-up for those getting revascularized will be 6 months and the maximum possible 24 months. Based on the previous studies by Radermacher, 20% of subjects with ARVD will have RRI ≥ 80[24]. It is unknown what fraction of those who have an indication for revascularization will have an RRI ≥ 80 but it is likely greater than 20%. An estimate of 1/3 of the revascularization group will be used. The numbers are likely to distribute as seen in Table 5. The purpose of the pilot is in part to determine this distribution of patients so that a large randomized study can be planned.

**Table 5:** Expected distribution of patients in study according to RRI status and need for revascularization assuming 240 patients screened

Total patients	240			
Requiring Renal revascularization	Yes (20%) 48		No (80%) 192	
RRI (estimated) N	< 80 (2/3) 32	≥ 80 (1/3) 16	< 80 (2/3) 128	≥80 (1/3) 64
Randomized to revascularization	Y/N 16/16	Y/N 8/8		

In the DRASTIC study, with follow up for one year, 8% of those randomized had elevations of creatinine by at least 50% [28]. The patients in the RAVE study are likely to have a greater rate of progression to end points as they will be a more severe group with ARVD. The DRASTIC study randomized to revascularization, all patients with renal vascular lesions > 50% with diastolic blood pressure > 95 mmHg on only 2 medications. Thus patients with isolated systolic hypertension or patients who would have responded to 3 or more medications were excluded in that study. Recruitment of patients for renal vascular disease can be difficult as demonstrated by the ongoing CORAL study with fewer than 25% enrolled more than 50% into the planned enrollment period. Indeed, in the first six months of enrollment fewer patients were seen in clinic than expected (40 vs 80) and less than 10% of these had indications for revascularization.

## Discussion

The RAVE study seeks to determine if a subset of patients with renovascular disease can be spared from invasive and expensive testing and possibly fruitless invasive procedures. If the renal resistance index can successfully identify those in whom microvascular disease has already damaged the affected kidney to the point that renal revascularization is not beneficial, it can serve a useful purpose in the management of this condition. The failure of previous revascularization studies to demonstrate a benefit may have been due to the inclusion of subjects with microvascular disease that would not benefit from treatment. This test if prospectively demonstrated to be of benefit may help to select a cohort of subjects that will benefit from further assessment and renal revascularization.

## Competing interests

The author(s) declare that they have no competing interests.

## Authors' contributions

SWT and CB were responsible for identifying the research question and drafting the study protocol. All authors have contributed to the development of the protocol and study desin, as members of the research team. SWT was responsible for drafting of this paper and all authors provided comments and have read and approved the final version.

**Table 3:** Study inclusion and exclusion criteria

Inclusion criteria:
• age 55 and older.
• systolic blood pressure > 140 mmHg and/or diastolic > 90 mmHg despite at least 3 antihypertensive medications.
• systolic blood pressure > 140 mmHg and/or diastolic > 90 mmHg on two antihypertensives with; a rise in creatinine > 20% after initiation of an ACEi or ARB
• the sudden onset of hypertension occurring after age 55
• hypokalemia
• the presence of an abdominal bruit
• history of flash pulmonary edema,
• three of; Peripheral vascular disease, coronary artery disease, cerebrovascular disease, smoking, hyperlipidemia, diabetes or male gender.
Exclusion Criteria:
• serum creatinine > 220 umol/L or estimated GFR by Cockroft Gault equation < 20 ml/min.
• patients who are unwilling or unable to give informed consent.
• Known contraindication to renal revascularization such as anaphylactic allergy to contrast dye
• an abdominal aortic aneurysm requiring surgery
• a single functioning kidney
• a total occlusion of the renal artery
• renal artery stenosis due to fibromuscular dysplasia
• previous revascularization.

## Pre-publication history

The pre-publication history for this paper can be accessed here:


